# Red Disperse Azo Dye Side Chains Influence on Polyethylene Terephthalate Dyeing Performances in Supercritical Carbon Dioxide Media

**DOI:** 10.3390/polym14245487

**Published:** 2022-12-15

**Authors:** Yu-Wen Cheng, Jean-Sebastien Benas, Fang-Cheng Liang, Shang-Ming Lin, Yu-Hang Huang, Wei-Wen Chen, Yu-Ting Chen, Chen-Hung Lee, Yang-Yen Yu, Chi-Ching Kuo

**Affiliations:** 1 Institute of Organic and Polymeric Materials, Research and Development Center of Smart Textile Technology, National Taipei University of Technology, Taipei 10608, Taiwan; 2 Department of Materials and Textiles, Asia Eastern University of Science and Technology, New Taipei City 220303, Taiwan; 3 Division of Cardiology, Department of Internal Medicine, Chang Gung Memorial Hospital-Linkou, Chang Gung University College of Medicine, Tao-Yuan 33305, Taiwan; 4 Department of Materials Engineering, Ming Chi University of Technology, New Taipei City 24301, Taiwan

**Keywords:** dyeing, supercritical carbon dioxide, end-chain structure, disperse azo dye, colorfastness

## Abstract

Supercritical carbon dioxide dyeing (SDD) as a dyeing media not only provides a friendly dyeing environment but also significantly increases polymeric dyeing performances ascribed to strong azo dye affinity. Disperse azo dyes have shown to be highly efficient dyeing agents due to their facile coupling synthesis, side chains position, and length tunability to optimize absorption properties. Herein, we first synthesize two series of disperse red azo dyes via a coupling chemical route. Further, we investigate the position of the electron withdrawing group and alkyl chains length impact onto the absorption and color fastness properties. Upon synthesis, ^1^H NMR and mass spectroscopy were used to characterize our newly synthesized series dye structure. Also, according to spectroscopic characterization, the functional group positions as well as the alkyl chains length have a major impact on the dye series maximum light absorption wavelength and performance. We have performed SDD dyeing of polyethylene terephthalate woven and determined each dye color fastness, we find that a reduced electron withdrawing effect and alkyl chains increase reduce color-fastness performances. Overall, our dyes exhibited a good resistance against detergent water, perspiration, abrasion, and friction.

## 1. Introduction

Water dyeing processes are a widely used chemical route to perform polymeric dyeing; however, water media for dyeing results in significant drawbacks including high chemical consumption, consequent resources costs, and various environmental problems. In addition, water usage may favor water–dye phase separation and water dye grafting at the surface of the polymeric materials [1,2,3,4]. To overcome these drawbacks, replacing water by supercritical carbon dioxide as the dyeing media brings serious benefits. Supercritical carbon dioxide dyeing offers numerous advantages such as highly limited environmental impact, mitigated polymer swelling, low surface viscosity favoring dyeing agent penetration, and diffusion with limited by-product formation [5,6,7,8,9,10,11,12,13]. Furthermore, it has been demonstrated that dyeing under supercritical carbon dioxide environment suppresses product denaturation and promotes dye-stuff recovery. As shown by Hou et al., supercritical carbon dioxide forms under a pressure of 7.38 MPa and a temperature of 31.1 °C, which favor a stable dyeing environment for the formation of a uniform dye phase and avoiding phase segregation between dye and host during dyeing [1]. Supercritical carbon dioxide has shown excellent performances for the dyeing of bio and synthetic polymer including wool [14], polyester [15], polystyrene [16], polyurethane [17,18], nylon [19,20], polyethylene terephthalate (PET) [13,15,21,22], and cotton [2,6]. During supercritical carbon dioxide dyeing (SDD), the free volume of fiber molecular chain expands, resulting in higher disperse dye penetration and diffusion without using secondary agents [21]. Numerous polymers interact with dye molecules via a network of H-bond and hydrophobic interaction facilitating extensive dye penetration. However, polyester such as PET exhibit a low density of dyeing site result in limited dye molecular diffusion within the polyester [23,24]. Simultaneously, most hydrophobic polymeric material employed for supercritical carbon dioxide dyeing exhibit acceptable dyeing affinity but their high glass transition temperature (Tg) further limit dyes diffusion through polymer amorphous area. By contrast, low-PET Tg (67–81 °C) significantly increase the amorphous ratio of the polymeric materials which result in facilitated processability as well as higher mass transfer rate within the PET materials [25,26]. Therefore, PET has become a material of promising interest for low-cost and high-efficiency dyeing processes with high affinity to azo disperse dye.

In the past decades, disperse azo dyes were employed as remarkable dyeing agent ascribed to their facile chemical route synthesis and peculiar structure. Common disperse azo dyes structure is constructed using an anthraquinone subunit as chromophore combined with nonionic monoazo functional group. The mechanism underlying light absorption is ascribed to the presence of a chromophore –N=N– group absorbing light in the visible range [27,28]. The facile synthesis of disperse azo dyes through a diazonium salt and coupling intermediate provides an easy pathway to modify dye structures, thereby, impacting their optical properties. According to Zhang et al., tuning the functional group of a given chemical structured enable to control the electron delocalization and profoundly affect the chromophore bandgap [29]. Coupling and diazonium salt precursor side chain chemical group are known to modify the resulting electron pushing–pulling equilibrium at the chromophore vicinity [3,30,31]. For instance, our group recently demonstrated that the position of electron withdrawing methoxy group on the diazonium salt provide a remarkable pathway to deepen the chromophore light absorption capability [13]; thereby translating a significantly high dye uptake within the dye host with a strong pulling effect from the chromophore [32,33]. A similar effect was observed by Zhang et al.; they observed improved dyes fastness using of aromatic hydroxyl group side chain disperse azo dye [3]. Therefore, the engineering of the diazonium salt electron withdrawing effect provided a viable strategy to synthesize dye with strong light absorption properties. It has also been found that alkyl chain length actively modifies chromophore bandwidth [34], increase dye affinity, but undergo crosslinking [35], thus modifying the dye absorption and electronic behavior. For instance, Pan et al. have found that alkyl chains hydrophobic interaction may locally increase polymer Tg and reduce chains dynamics. A locally thickened polymer is likely to reduce dye molecule insertion and diffusion within the polymeric host [36]. Therefore, both end-chain alkyl chains length and the electro-active group plays a significant role for light absorption and dye uptake performances.

Herein we fabricated two dyes series, namely dye 161 series (3-(N, N-diethyl)aminoacetaniline) and dye X-377-X-D (3-amino-4-methoxyacetaniline). We optimized the position of the dye 161 methoxy group to maximize the electron withdrawing effect, whereas the dye X-377-X-D alkyl chains length was increased from two to eight carbon long to study the impact of hydrophobic forces. Both dye series structure was assessed by mass spectroscopy (MS) and nuclear magnetic resonance (NMR) while optical performances were determined by ultraviolet-visible (UV-Vis) optical characterization. To further assess dyes series color fastness and the impact of their structure, we have employed PET as a dyeing host. We have sewed various fabrics to determine our dye color fastness in water, detergent, perspiration, and upon abrasion and friction. We have observed that the methoxy group position of the diazonium salt enable to shift our dye light absorption wavelength, while alkyl chains length modification plays a major role in dyeing and color-fastness performances. Overall, we find that our disperse azo dyes in PET meet and surpass commercial standard in various environment.

## 2. Experimental Process

### 2.1. Materials

Diazonium salt precursors: Aniline, 4-Methoxy-2-nitroaniline, 4-Nitroaniline, 3-amino-4-methoxyacetanilide, and 5-Methoxy-2-methyl-4-nitroaniline were purchased from Sigma-Aldrich (St. Louis, MO, USA). All purchased products were used as received for dye synthesis.

Dye coupling precursors: 3-(N,N-diethyl)amino acetaniline, 3-(N,N-dihexyl)amino-4-methoxyacetaniline, and 3-(N,N-dioctyl)amino-4-methoxyacetaniline were purchased from Sigma-Aldrich and used as received for dye synthesis. 3-(N,N-dibutyl)amino-4-methoxyacetaniline, 3-(N,N-diethyl)amino-4-methoxyacetaniline were synthesized and characterized in our laboratory.

Chemicals: Sodium nitrite, cellulose acetate, ethyl acetate, sodium carbonate, isopropanol, acetone, sulfamic acid, sodium acetate, hydrochloric Acid (37%), toluene, ethanol, and n-hexane were purchased from Taiwan Green Version Technology Ltd. (New Taipei City, Taiwan) and used as received. Sodium hydroxide was purchased from Sigma-Aldrich (USA) and used without further purification. Thin-film for chromatographic analysis was bought from Macherey-Nagel Polygram^®^ SIC G/UV 254 (Düren, Germany). Carbon dioxide (CO_2_) was obtained from Banqiao Gas Co., Ltd. (Banqiao Gas Co., Ltd, New Taipei City, Taiwan). In addition, 100% cotton plain weave and knitted Polyester 75D/72F fabricated from 100% polyester fabric were supplied by Far Eastern New Century (Taipei, Taiwan). White cotton fabric for AATCC friction fastness test, WOB lotion and DW multi-fiber fabric for SDC ISO standard and stainless-steel beads with corrosion resistance properties were provided by Gao Yi Enterprise Co., Ltd. (Kaohsiung City, Taiwan).

### 2.2. Preparation of Dye

#### 2.2.1. Diazonium Solution Preparation

A certain amount of aniline diazonium precursor (Table S1) with 10 mL of ultrapure water was added into a beaker. Further, 6 mL of hydrochloric acid (37%) was swiftly poured into the stirring solution under heating. Following precursor dissolution, ice cubes were added into the solution until the temperature reached 0–5 °C. Upon cooling, 0.76 g (0.011 mol) of sodium nitrite aqueous solution was steadily poured under stirring. The solution yellow color disappearance upon stirring translates a terminated diazotization reaction. 4-diethyl Aminobenzaldehyde (IP Solution) is used to control yellow color intensity, indicating the presence of sulfamic acid. If nitrous acid is being sensed by potassium iodide paper test, sulfamic acid is removed until complete sulfamic acid trace signal disappearance. Therefore, a light yellow turbid-free solution shows a successful diazonium solution synthesis.

#### 2.2.2. Coupling Component Synthesis

For dye coupling component preparation, 18.02 g (0.1 mol) of 3-amino-4-methoxyacetanilide was added with 108 mL of an aqueous solution, 45 mL of isopropanol, and 53 g (0.5 mol) of sodium carbonate in a three-necked flask. The mixture was heated to 80 °C, and stirred for 60 min. Upon the controlled addition of 53 g (0.5 mol) of 1-iodobutane through a separatory funnel, the mixture was refluxed and reacted for 8 h. Upon reaction completion, the 1-iodobutane excess and isopropanol were removed by vacuum distillation. Further, 100 mL of water and 50 mL of ethyl acetate were added to the residue and have undergone phase separation after 1 h rest. A separatory funnel was used to separate and remove the water layer with anhydrous sodium sulfate. The resulting mixture was filtered and distilled under reduced pressure to obtain a dark brown oily 3-(N,N-Dibutylamino)-4-methoxyacetaniline. Similarly, the 1-iodobutane is changed to 1-iodoethane to obtain the 3-(N,N-diethylamino)-4-methoxyacetaniline intermediate for further dye fabrication.

#### 2.2.3. Dye Synthesis

A coupling agent is added to a water–cellulose acetate mixture solution of 1:3 *v*:*v* (mL) (2.5:7.5 mL) at 0–5 °C. The diazonium solution is gently poured into the coupling component solution at the above temperature under vigorous stirring until the reaction is achieved. Further, sodium acetate is added to regulate the pH of the solution slightly acidic. In order to set the dye pH at a neutral pH after dye precipitation and filtration, ultrapure water was used. Upon filtering, TLC was performed to detect impurity presence within the dye in a toluene: ethyl acetate: cellulose acetate = 8:2:1 solution. Upon impurities detection, a washing step with ethanol was conducted and followed by filtration until complete removal of impurities. Each dye synthesis follows the same chemical route with appropriately selected diazo and coupling component. Final structures and synthesis parameters are shown in Table 1 and Table S1.

### 2.3. Dye Characterization

Purified dyes structures were characterized using MS, and ^1^H-NMR in Tetramethylsilane (TMS) reference (Bruker Advance III HD-600 MHz liquid, Bruker Daltonik GmbH, Bremen, Germany). For ^1^H-NMR measurement, 10 mg of dye was mixed with 1 mL of CDCl_3_, further poured into a ^1^H-NMR tube. 

### 2.4. Dyeing Apparatus

The supercritical carbon dioxide dyeing chamber is illustrated in Figure S1. An amount of 20 mg polyester fabric of 75 D/72 F size was embedded with a 1% dye solution. The sample was then into the SDD machine chamber for a duration dye time of 60 min with a pressure and temperature of 3625 psi and 120 °C, respectively.

### 2.5. Color Fastness Performance Characterization

The dye K/S color fastness is extracted from the Kubelka and Munk formula (1) [37]:

(1) $$\frac{K}{S}=\frac{{\left(1-R_{\infty }\right)}^{2}}{2R_{\infty }}$$

S is the back-scattering coefficient, K is the absorption and R_∞_ the re-emission fraction of an incident light for an infinite thickness layer. Dyes relative coloring rate is calculated assuming dyes sample are exhaustion-free during supercritical dyeing. Upon a continuous dyeing process, dye exhaustion enables to calculate the relative coloring rate. The first K/S is set as the first dyeing cycle and further divided by accumulated K/S value extracted from continuous dyeing cycle. The term ${\left(\frac{K}{S}\right)}^{\mathrm{St}}$ represents the first dyeing K/S and $\overset{\infty }{\underset{n=1}{\sum }}{\left(\frac{K}{S}\right)}_{n}$ represent the successive K/S value accumulation for continuous dyeing cycle as shown below:

(2) $$\mathrm{Relative}\;\mathrm{coloring}\;\mathrm{rate}\;=\frac{{\left(\frac{K}{S}\right)}^{\mathrm{st}}}{\sum_{n=1}^{\infty }{\left(\frac{K}{S}\right)}_{n}}$$

### 2.6. Dyeing Fastness Test

According to the ISO 105 C06: 2010 procedure, polyester, cellulose acetate, acrylic, nylon, wool, and cotton were cut together into a 10 cm × 4 cm fabric to undergo fastness tests with dyed material. Fabricated products were placed within a stainless-steel bottle with the following dimension: volume: 550 mL, height: 125 mm, diameter: 75 mm. Further, the bottle was deposited into a washing test apparatus alongside the AATCC 1993 Standard Detergent (WOB), ultrapure water as well as 50 steel balls. The washing test apparatus was set at 40 ± 2 rpm and remained constant during the washing test. Upon washing completion, the sample was further washed under a temperature of 40 °C during 1 min with 100 mL of ultrapure water. Upon washing completion, fabrics were separated to perform drying at a temperature below 60 °C.

### 2.7. Water Fastness Test

Based on the ISO 105-E01 procedure, 10 cm × 4 cm cloth fabric polyester, cellulose acetate, acrylic, nylon, wool and cotton were cut together with dyed material to undergo fastness tests. Then, samples were homogeneously dipped at ambient temperature for 30 min within ultra-pure water. Further, the sample was surrounded and pressed by two glasses in order to perform water removal. Upon water removal, the fabric was positioned onto an acrylic resin plate with an applied load of 5 kg within a preheating test apparatus. The apparatus temperature was fixed at 37 °C to conduct water fastness test for 4 h. Upon washing completion, fabrics were separated to perform drying at a temperature below 60 °C.

### 2.8. Perspiration Fastness Test

According to the ISO 105-E04 specifications, acid and alkali media were used to replicate sweat effect, following test were performed following the above procedure. 

### 2.9. Abrasion and Rubbing Color Fastness Test

An AATCC standard white cotton fabric was fixed into the cylindrical friction head to undergo dry friction test. The friction head downward pressure is set to 9N and configured to perform 10 cycles of back and forth scrubbing at 1 time per second. For the wet friction procedure, an AATCC standard white cotton fabric was humidified (65% (±5%)) at ambient temperature using an absorbent paper. The humidified sample was clipped onto the cylindrical friction, and then placed onto the machine sandpaper. The friction head downward pressure was set to be 9N and configured to perform 10 cycles at 1 cycle/s. Upon the cycle’s completion, both test cotton fabrics were separated to perform drying at a temperature below 60 °C in air. All fastness tests results are validated upon evaluating color change or gray scale of the cottons fabric.

## 3. Results and Discussion

### 3.1. Dyes Synthesis and Characterization

**Figure 1:**
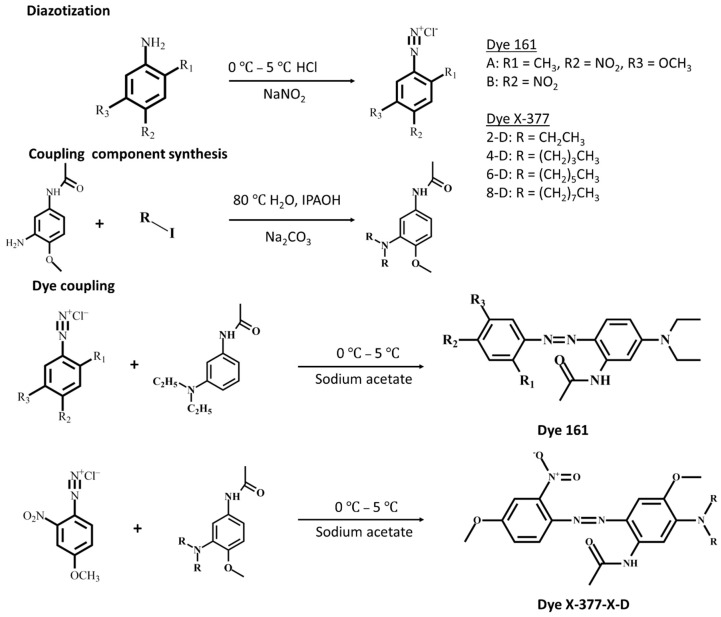
Chemical route of diazotization reaction, coupling component synthesis of dye precursor and coupling reaction between diazotized salt and coupling component.

Disperse azo dyes series were synthesized using a coupling component and a diazonium salt, illustrated in Figure 1 while structure and yield synthesis are summarized in Table 1. Dye series 161 diazonium salt precursor was synthesized at 0–5 °C by diazotization reaction in which the aromatic primary amine (2-Methoxy-4-nitroaniline, 5-Methoxy-2-methyl-4-nitroaniline or 4-Nitroaniline) was functionalized by side-ring chemical function and underwent deprotonation to obtain a diazonium cation. Dye X-377-X-D series diazonium salt (4-Methoxy-2-nitroaniline) was synthesized following a chemical route similar to the dye series 161. Dye X-377-2-D (3-(N,N-diethyl)amino-4-methoxyacetaniline) and dye X-377-4-D (3-(N,N-dibutyl)amino-4-methoxyacetaniline) coupling component were synthesized from 3-amino-4-methoxyacetanilide (Figure 1). Both coupling components were characterized through MS and ^1^H-NMR for both chemical structures. 3-(N,N-dibutyl)amino-4-methoxyacetaniline with a C_17_H_28_N_2_O_2_ structure MS analysis is given as follows. HRMS, m/z is determined for [M^+^], 292.2 (100%) as the reference; m/z: determined for [M–COCH_3_], 249.2 (50%) exhibiting an ionic peak, originating from –COCH_3_ groups presence. ^1^H-NMR spectroscopy data shows (Figure S2) the presence of a double hydrogen peak of hydrogen with a δ7.09 chemical shift, ascribed to the hydrogen on the carbon a of the benzene ring. Furthermore, a single peak of hydrogen is observed at δ7.07, corresponding to the hydrogen on carbon c of the benzene ring. The double peak of hydrogen at δ6.76–6.74 chemical shift highlights the hydrogen on carbon b of the benzene ring. In addition, three single hydrogen peaks were observed at δ3.78 position, corresponding to the hydrogen on carbon d. The presence of four triple hydrogen peaks at δ3.06–δ3.03 translates the presence of the hydrogen on carbon f. Moreover, three single-hydrogen peaks at δ2.11 correspond to the hydrogen carbon e. Four multiple hydrogen peaks at the position of chemical shift δ1.4–1.38 are associated to the hydrogen on carbon g1. Four multi-wave peaks of hydrogen at the δ1.24–1.22 chemical shift are ascribed to the hydrogen on carbon g2. Finally, six hydrogen peaks at the chemical shift δ0.87–δ0.84 position are ascribed to the hydrogen on the h carbon. Both MS and ^1^H-NMR indicate the successful synthesis of the 3-(N,N-diethyl)amino-4-methoxyacetaniline and 3-(N,N-dibutyl)amino-4-methoxyacetaniline coupling component.

Upon completion of the diazonium salt synthesis, six disperse azo dye were fabricated through a facile coupling chemical process. It is commonly admitted that the coupling process occurs under acidic conditions to increase the dye stability, as well as at a temperature of 0–5 °C to maintain the diazonium salt stability [3,38]. Furthermore, the choice of the functional group of the diazonium salt aromatic rings influence the rate of the coupling reaction in which a strong electron-withdrawing function will increase the coupling kinetics, resulting in decreased salt stability [3,13,27]. The dyes series 161 were obtained from previously obtained diazonium salt with the following 3-(N,N-diethyl)amino acetaniline coupling component. An identical approach was undergone for the dye series X-377-X-D via the coupling of diazonium salt (4-Methoxy-2-nitroaniline) and the following coupling component (3-(N,N-diethyl)amino-4-methoxyacetaniline, 3-(N,N-dioctyl)amino-4-methoxyacetaniline, 3-(N,N-dibutyl)amino-4-methoxyacetaniline and 3-(N,N-dihexyl)amino-4-methoxyacetaniline). Figure 1 illustrates two derivates of dye 161 and four derivates of dye X-377-X-D. We have designed our dyes in a D-π-A model to promote an efficient electron transfer toward the azo groups. The azo group acts as the chromophore while the diazonium salt withdraws electrons from the chromophore. Simultaneously, the coupling component provide electron to the chromophore. The dye 161 series was investigated according to the position of their functional group on the diazonium salt while the dye X-377-X-D alkyl chain length effect of the coupling component was investigated. The resulting dyes structural properties as well as their specific impact onto their optical and dyes are thoroughly investigated below with the dye lettering indicated in the Figure S3. Figures S4–S9, Tables S2 and S3 display dye 161 and X-377-X-D series MS analysis. Dye 161-A with a C_20_H_25_N_5_O_4_ structure MS analysis is given as follows. HRMS, m/z is determined for [M^+^], 399.2 (100%) as the reference; m/z: determined for [M–CH_3_], 384.2 (55%) exhibited an ionic peak, originating from –CH_3_ groups presence; m/z: determined for [M–COCH_3_], 369.3 (40%) has an ionic peak, originating from two –CH_3_ groups presence. Dye 161-B with a C_18_H_21_N_5_O_3_ structure MS analysis is given as follows. HRMS, m/z is determined for [M^+^], 355.2 (100%) as the reference; m/z: determined for [M–2CH_3_], 340.1 (50%) exhibited an ionic peak, originating from –CH_3_ groups presence; m/z: determined for [M–COCH_3_], 325.2 (40%) has an ionic peak, originating from two –CH_3_ groups presence (Figures S4 and S5). Dye X-377-2-D with a C_20_H_25_N_5_O_5_ structure MS analysis is given as follows. HRMS, m/z is determined for [M^+^], 415.2 (100%) as the ionic reference peak; m/z: determined for [M^+^–CH_3_], 400.2 (98%) exhibited an ionic peak, originating from –CH_3_ groups presence; m/z: determined for [M^+^-N=N-Ar-2-NO_2_-4-OCH_3_], 235.2 (15%) has an ionic peak, originating from –C_13_H_17_N_2_O groups presence. Dye X-377-4-D with a C_24_H_33_N_5_O_5_ structure MS analysis is given as follows. HRMS, m/z is determined for [M^+^], 471.3 (67%) as the ionic reference peak; m/z: determined for [M^+^–CH_2_CH_2_CH_3_ or M^+^–COCH_3_], 428.3 (100%) exhibited an ionic peak, originating from one –CH_2_CH_2_CH_3_ or –COCH_3_ group presence; m/z: determined for [M^+^–5CH_3_], 396.3 (43%) has an ionic peak, originating from five –CH_3_ groups presence. Dye X-377-6-D with a C_28_H_41_N_5_O_5_ structure MS analysis is given as follows. HRMS, m/z is determined for [M^+^], 527.3 (57%) as the reference; m/z: determined for [M^+^– C_5_H_11_], 456.2 (85%) exhibited an ionic peak, originating from –CH_2_CH_2_CH_2_CH_2_CH_3_ groups presence; m/z: determined for [M^+^-N=N-Ar-2-NO_2_-4-OCH_3_-COCH_3_ -C_6_H_13_], 369.3 (40%) exhibited an ionic peak comparable to the reference signal, originating from –C_13_H_17_N_2_O groups presence. Dye X-377-8-D with a C_32_H_49_N_5_O_5_ structure MS analysis is given as follows. HRMS, m/z is determined for [M^+^], 583.6 (80%) as the reference; m/z: determined for [M^+^–C_7_H_15_], 384.2 (55%) exhibited an ionic peak, originating from –CH_2_CH_2_CH_2_CH_2_CH_2_CH_2_CH_3_ groups presence; m/z: determined for [M^+^-Ar-2-NO_2_-4-OCH_3_-C_7_H_15_C_8_H_17_ or M^+^-N=N-Ar-2-NO_2_-4-OCH_3_-C_5_H_11_-C_8_H_17_], 219.2 (20%) exhibited an ionic peak comparable to the reference signal, originating from two –CH_3_ groups presence (Figures S6–S9).

Dye 161 and X-377-X-D structural characterization is given by ^1^H-NMR spectroscopy and illustrated in Figures S10–S15. The ^1^H-NMR spectrum data of dye 161-A shows a triplet peak of six hydrogens with a chemical shift of δ1.335~1.359, which could be the hydrogen localized on the N-alkane chain carbon g. Furthermore, three hydrogens single peak exhibited at δ2.537, which is ascribed to the acetyl carbon e hydrogen of the dye. Three hydrogens single peaks at the δ2.658 chemical shift are determined to be the methyl carbon 1d. There are four hydrogens quartet peak at the position δ3.569~3.605, and correspond to hydrogen localized on the carbons f of the dye N-alkane chain. The chemical shift of δ4.018 is associated to a three single peaks of hydrogens, ascribed to the carbon 1b hydrogen. The presence of a hydrogen single peak at the position δ7.573, ascribed to the carbon b hydrogen. In addition, two hydrogen double peaks at δ7.738~7.745 corresponds to the hydrogen on carbon a and c. A single peak of hydrogen found at δ7.858 chemical shift is considered to be the carbon 1c hydrogen. Finally, a single peak of hydrogen at δ8.528 chemical shift, is associated to the carbon 1a hydrogen. Others dyes series ^1^H-NMR studies are summarized in the Table S4. To conclude, MS and ^1^H-NMR characterization confirm the integrity of our synthesized dye structures and the influence of functional group and side chains will be investigated further.

### 3.2. Dye Optical Characterization

Disperse azo dyes D-π-A model enable dyes to exhibit deep and tunable color of emission via the nature of the functional group grafted on the diazonium and coupling component. Based on their structure and electronegativity, each specific chemical function will exhibit an electron-donor or withdrawing effect that will significantly impact the resulting absorption properties. In order to determine our dyes series spectroscopic response, UV-Vis experiment was performed and the maximum absorption wavelength (λ max) as well as the absorption coefficient (ε) were extracted (Table 2). Accordingly, Dyes 161 series exhibit a shift from the 503 to the 511 nm region upon electron withdrawing methoxy group removal from the diazonium salt meta-position, hence reducing the electron-withdrawing effect from the chromophore. As observed for the dye X-377-X-D series, the alkyl chains length has no significant effect on the position of the maximum of absorption while the methoxy group electron delocalization effect is weaker due to its position at the para position, resulting in a wavelength blue shifting. However, we find that ε is ranging from 82,000 L·mol^−1^·cm^−1^ up to 87,000 L·mol^−1^·cm^−1^. The influence of the methoxy group position is significant for the dye 161 series, the loss of the withdrawing effect significantly reduces the light absorption capabilities as the dye 161-B ε value is smaller than the dye 161-A. The weakening of the electron-withdrawing effect may reduce the hypsochromic strength [33]. In addition, the X-377-X-D dye series ε decreased significantly with the increase in the alkyl chain length. We find that ε is ranging from 14,000 L·mol^−1^·cm^−1^ up to 92,000 L·mol^−1^·cm^−1^ with alkyl chain length shortening. This may be attributed to dye aggregation caused by alkyl chains crosslinking [35]. In addition, the alkyl chains length increase may offset the electron withdrawing effect of diazonium component while decreasing the electron donating effect of the coupling component. Hence, our dyes long alkyl chains effect results in degraded electron transport from the donor moieties toward the chromophore. Herein, the position of the functional group impacts the absorption capability of our dye series. Also, we find that alkyl chains play a significant role within the disperse azo dyes D-π-A model absorption capabilities but longer alkyl chains may decrease dyes light absorption performances.

### 3.3. Dyeing Performances

#### 3.3.1. Apparent Colorfastness K/S 

In order to overcome the water polyester dyeing hindrance including pre-treatment and auxiliary agents coating, SDD enable effective dispersal of azo dye dyeing and suppressed CO_2_-dye phase separation. Dispersed azo dyes were blended with the PET fabric at 1% concentration to undergo a SDD process of a 60 min duration at 120 °C. During SDD process, the higher temperature increased dye penetration kinetics through PET fibers amorphous area due to the PET chain softening and amorphous pore dimension increase. During the dyeing process, hydrophobicity imbalance blocked CO_2_ penetration into the PET fabric, thus, promoting proper dye diffusion within the PET material through triggering intermolecular interaction between PET and the dispersed azo dye. In addition, large amorphous ratio of PET significantly increases dye diffusion kinetics [39,40]. 

The dye absorption efficiency was extracted according to the K/S formula, we find that the color density varies from 3.2 (Dye X-377-8D) to 21.0 (Dye 161-A) (Table 3). We find that dye relative coloring rate increased in correlation with the dye absorption efficiency. The dye 161-A first relative coloring rate reached 88.82% and is higher than other dye series. First, we observed that the electron withdrawing effect of the methoxy group significantly improved the color fastness as shown by the dye 161-A series dye (hypsochromic effect) [33]. Second, we observed a significant decrease in the dye X-377-X-D color fastness in correlation with the light absorption weakening. In the literature, it has been shown that the alkyl chains length grafted onto the phenyl ring improved dyeing fastness performances without impacting the maximum absorption [41]. Moreover, alkyl chains side chains with increased length reduced dye diffusion within polymeric pores [42]. We assumed that the increased alkyl chains significantly increased the dyeing molecule size and mitigating the dye diffusion through PET amorphous pores. Also, excessive length of carbon chains may increase SDD-disperse dye hydrophobic interaction, which may decrease dye uptake. Therefore, shorter lengths of alky chain length side chains significantly improved disperse azo dyes dyeing efficiency and color fastness.

#### 3.3.2. Color Fastness Properties

Based on ISO test regulation, dyes washing and fastness are organized on 1 to 5 scale, and high washing fastness output is usually classified with 4–5 grade. As described in the experimental method, cotton, wool, acrylic, nylon, polyester, and cellulose acetate fabric were fixed with dyed polyester textile to determine the dyes’ resilience against various environment. First, upon exposition to a standard detergent, dye 161-A, dye X377-2-D, and X-377-4-D washing fastness are classified as grade 2 when sewed to the nylon fabric. Others dyes fixed with all fabrics reach grade 2–3 and above. The dye X377-8-D exhibits a detergent washing fastness above grade 4 for all fabric, which meets the commercial standard (Table S5). Furthermore, all fabrics were exposed to highly pure water before being dried as indicated by the ISO 105 E01:2010 requirement (Table 4). For all fabrics, commercial standards are reached as fastness to water is equal to or above grade 3. Besides water fastness, resistance to alkali (Table S6), and acid (Table 5) environment were determined and we found that the color fastness is above 4 for all fabrics. Accordingly, the dye X377-8-D exhibits the best performances against water and sweat environment fastness. Finally, abrasion and rubbing dyeing fastness tests show that the dye X377-8-D exhibits a dyeing fastness against abrasion of 3–4 and above 4 for rubbing test. All of others dye fabrics are above 4 for both test and reach commercial standards (Table 6). We find that dyes uptakes and diffusion is strong enough to enable efficiency dyeing fastness performances and meet current commercial standards.

## 4. Conclusions

Herein, six disperse azo dyes were synthesized via coupling between a diazonium salt and coupling component and were characterized by ^1^H-NMR and MS and enable efficient PET dyeing in supercritical dioxide carbon media. By tuning the nature of the chemical function of the diazonium salt, the dye 161 series maximum absorbance was set at the 503–511 nm region. Furthermore, the absorbance was significantly impacted due to the meta-position methoxy group of the diazonium salt. Simultaneously, the increased alkyl chains length of the dye X-377-X-D series is correlated with lower absorption and reduced relative coloring rate. Upon performing SDD, all dyes series were tested for their fastness against detergent, water, alkali, acid environments, as well as an abrasion and rubbing test. The majority of synthesized disperse azo dyes can diffuse efficiently within the PET and reach the commercial fastness standard.

## Figures and Tables

**Table 1:** Synthesized dye general structure and yield.

Dye	R_1_	R_2_	R_3_	General Structure	Mass	Molecular Formula	Yield
161-A	CH_3_	NO_2_	OCH_3_	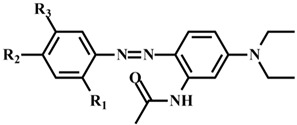	399.19	C_20_H_25_N_5_O_4_	78.7%
161-B	-	NO_2_	-		355.16	C_18_H_21_N_5_O_3_	81.6%
**Dye**	**R**			**General Structure**	**Mass**	**Molecular** **Formula**	**Yield**
X-377-2-D	C_2_H_5_			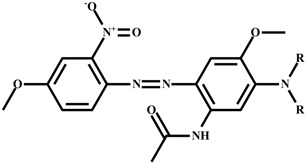	415.19	C_20_H_25_N_5_O_5_	82.6%
X-377-4-D	C_4_H_9_				471.25	C_24_H_33_N_5_O_5_	77.2%
X-377-6-D	C_6_H_13_				527.31	C_28_H_41_N_5_O_5_	76.3%
X-377-8-D	C_8_H_17_				583.37	C_32_H_9_N_5_O_5_	62.5%

**Table 2:** Series dyes optical characterization.

Dye	Maximum Absorption Wavelength (nm)	Absorbance	Absorption Coefficient (L·mol^−1^·cm^−1^)	Log (e)
161-A	503	0.870034	87,003.4	4.939
161-B	511	0.828747	82,874.7	4.918
X-377-2-D	501	0.929598	92,959.8	4.968
X-377-4-D	503	0.649098	64,909.8	4.812
X-377-6-D	502	0.321163	32,116.3	4.506
X-377-8-D	502	0.143213	14,321.3	4.155

**Table 3:** Series dyes supercritical carbon dye apparent color concentration and first time dyeing relative color.

Dye	Apparent Color Density	Relative Color First Time
161-A	21.0380	87.07%
161-B	13.51	72.33%
X-377-2-D	17.8810	83.23%
X-377-4-D	15.6820	75.32%
X-377-6-D	9.3760	67.03%
X-377-8-D	3.2750	65.21%

**Table 4:** Post-dyeing dye pure water fastness under the ISO 105 E01:2010 protocol.

Dye	Cellulose Acetate	Cotton	Nylon	Polyester	Acrylic	Wool
161-A	4	4–5	3–4	4–5	5	5
161-B	4–5	4–5	3–4	4–5	5	5
X-377-2-D	4	4–5	3–4	4–5	5	5
X-377-4-D	4–5	4–5	4	5	5	4–5
X-377-6-D	4–5	4–5	4–5	4–5	5	5
X-377-8-D	5	5	5	5	5	4–5

**Table 5:** ISO 105 E04:2008 protocol acidic media color fastness.

Dye	Cellulose Acetate	Cotton	Nylon	Polyester	Acrylic	Wool
019-A	4–5	5	4–5	5	5	5
019-B	4–5	4–5	4	4–5	5	5
X-377-2-D	4–5	4–5	4	4–5	5	5
X-377-4-D	4–5	5	4–5	5	5	5
X-377-6-D	4–5	4–5	4–5	4–5	5	5
X-377-8-D	5	5	5	5	5	4–5

**Table 6:** AATCC 8 protocol abrasion and rubbing color fastness.

Dye	Dry Friction	Wet Friction
019-A	4	4–5
019-B	4	4–5
X-377-2-D	4	4–5
X-377-4-D	4	4–5
X-377-6-D	4–5	4–5
X-377-8-D	3–4	4–5

## Data Availability

Data presented in this study are available on request from the corresponding author.

## Supplementary Materials

The following supporting information can be downloaded at: https://www.mdpi.com/article/10.3390/polym14245487/s1, Figure S1: Supercritical carbon installation for disperse azo dye dyeing; Figure S2. Synthesized coupling component and coupling component 3-(N,N-Dibutylamino)-4-methoxyacetaniline lettering for NMR characterization; Figure S3: Dye series lettering for NMR characterization; Figure S4: Dye 161-A mass spectroscopy analysis; Figure S5: Dye 161-B mass spectroscopy analysis; Figure S6: Dye X-377-2-D mass spectroscopy analysis; Figure S7: Dye X-377-4-D mass spectroscopy analysis; Figure S8: Dye X-377-6-D mass spectroscopy analysis; Figure S9: Dye X-377-8-D mass spectroscopy analysis; Figure S10: Dye 161-A ^1^H-NMR analysis in CDCl_3_; Figure S11: Dye 161-B ^1^H-NMR analysis in CDCl_3_; Figure S12: Dye X-377-2-D ^1^H-NMR analysis in CDCl_3_; Figure S13: Dye X-377-4-D ^1^H-NMR analysis in CDCl_3_; Figure S14: Dye X-377-6-D ^1^H-NMR analysis in CDCl_3_; Figure S15: Dye X-377-8-D ^1^H-NMR analysis in CDCl_3_; Table S1: Experimental parameters of disperse dye synthesis; Table S2: Mass spectrum analysis of dye 161 series; Table S3: Mass spectrum analysis of dye X-377-X-D series; Table S4: Disperse dye series NMR analysis; Table S5: Washing fastness to detergent after dyeing according to the ISO 105 C06:2010 protocol; Table S6: Color fastness in alkali media after dyeing according to the ISO 105 E04:2008 protocol.
