# Supplementary material for: Red Disperse Azo Dye Side Chains Influence on Polyethylene Terephthalate Dyeing Performances in Supercritical Carbon Dioxide Media
Source: Polymers (Basel). 2022 Dec 15;14(24):5487. doi: 10.3390/polym14245487 (PMC9782905; doi:10.3390/polym14245487)
Supplement: Supplementary file 1 [file polymers-14-05487-s001.zip › polymers-2090299-SI.pdf]

Supplementary Materials

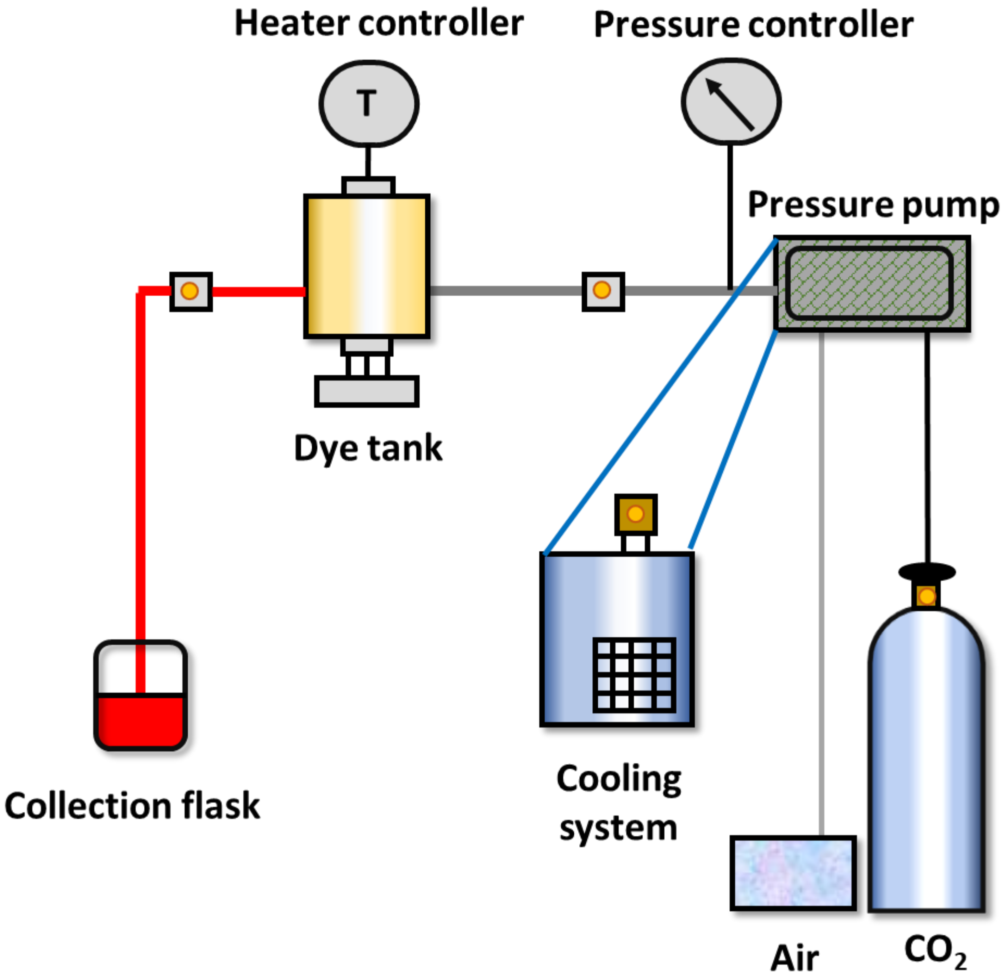

Figure S1. Supercritical carbon installation for disperse azo dye dyeing.

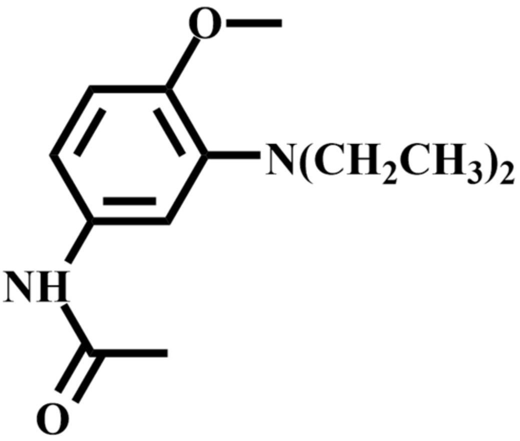

3-(N,N-Diethylamino)-4-methoxyacetaniline

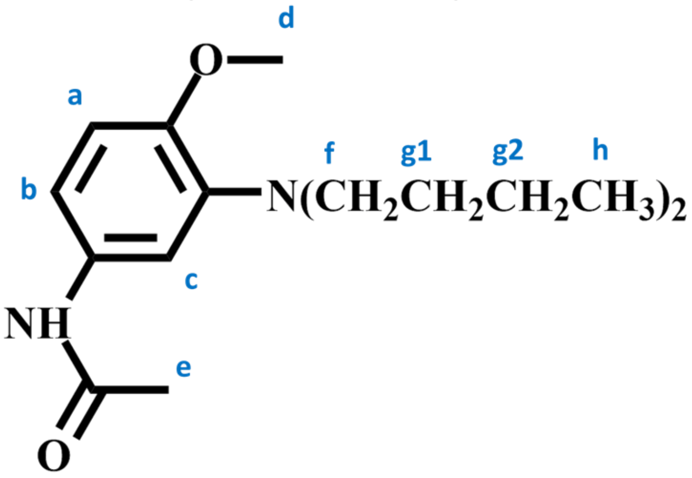

3-(N,N-Dibutylamino)-4-methoxyacetaniline

Figure S2. Synthesized coupling component and coupling component 3-(N,N-Dibutylamino)-4-methoxyacetaniline lettering for NMR characterization.

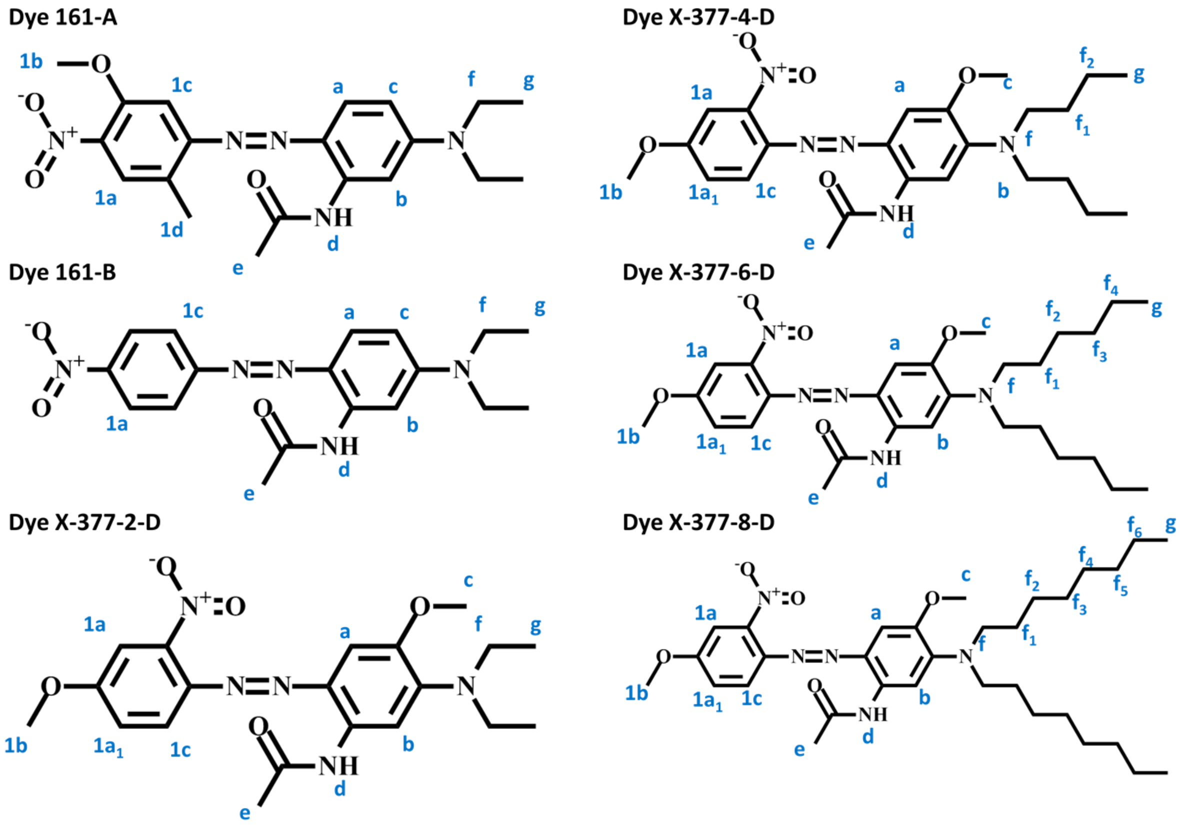

Figure S3. Dye series lettering for NMR characterization.

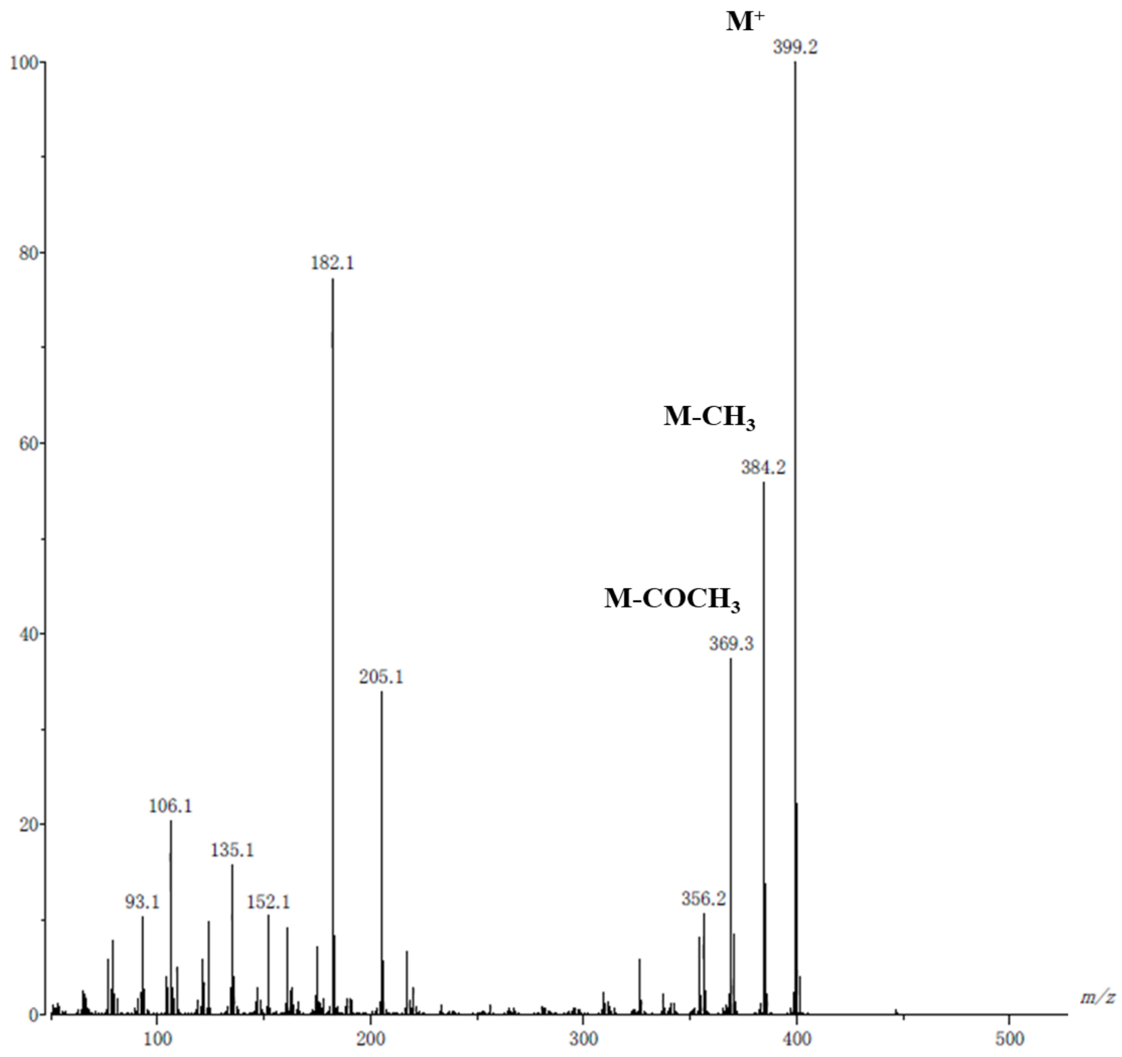

Figure S4. Dye 161-A mass spectroscopy analysis.

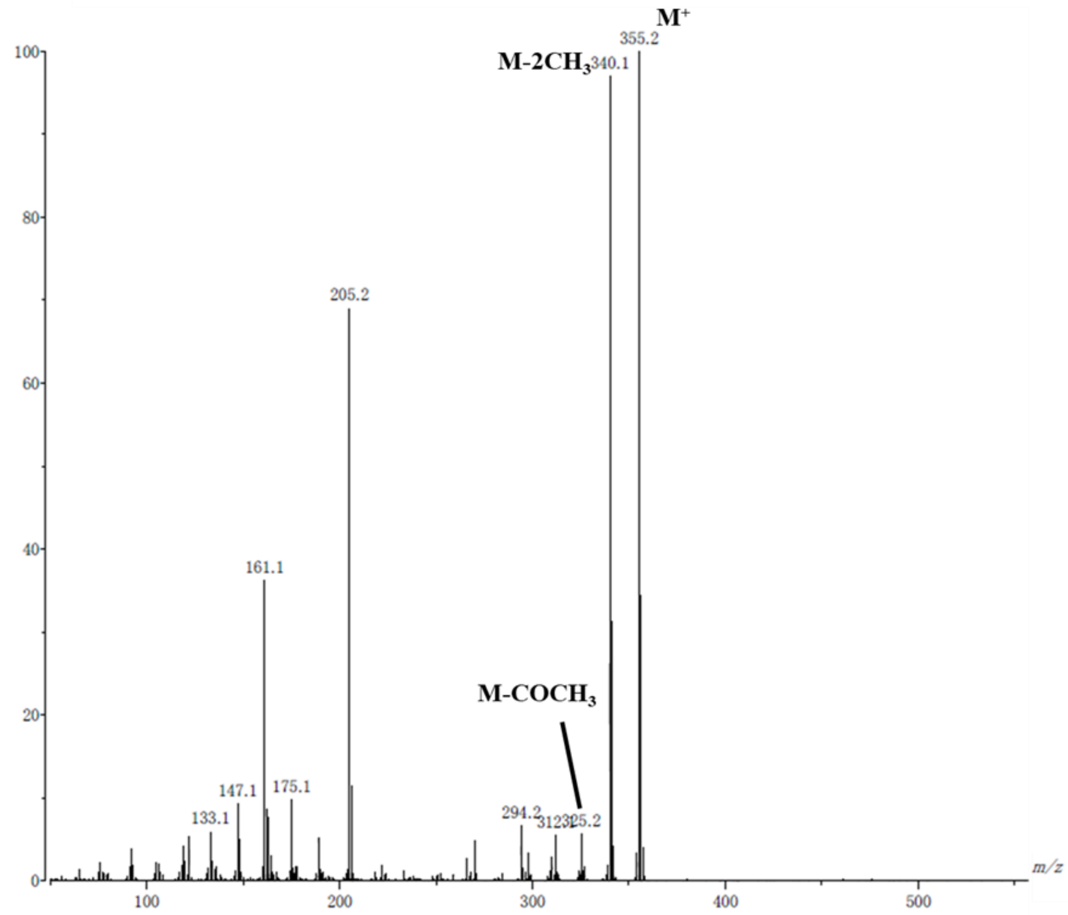

Figure S5. Dye 161-B mass spectroscopy analysis.

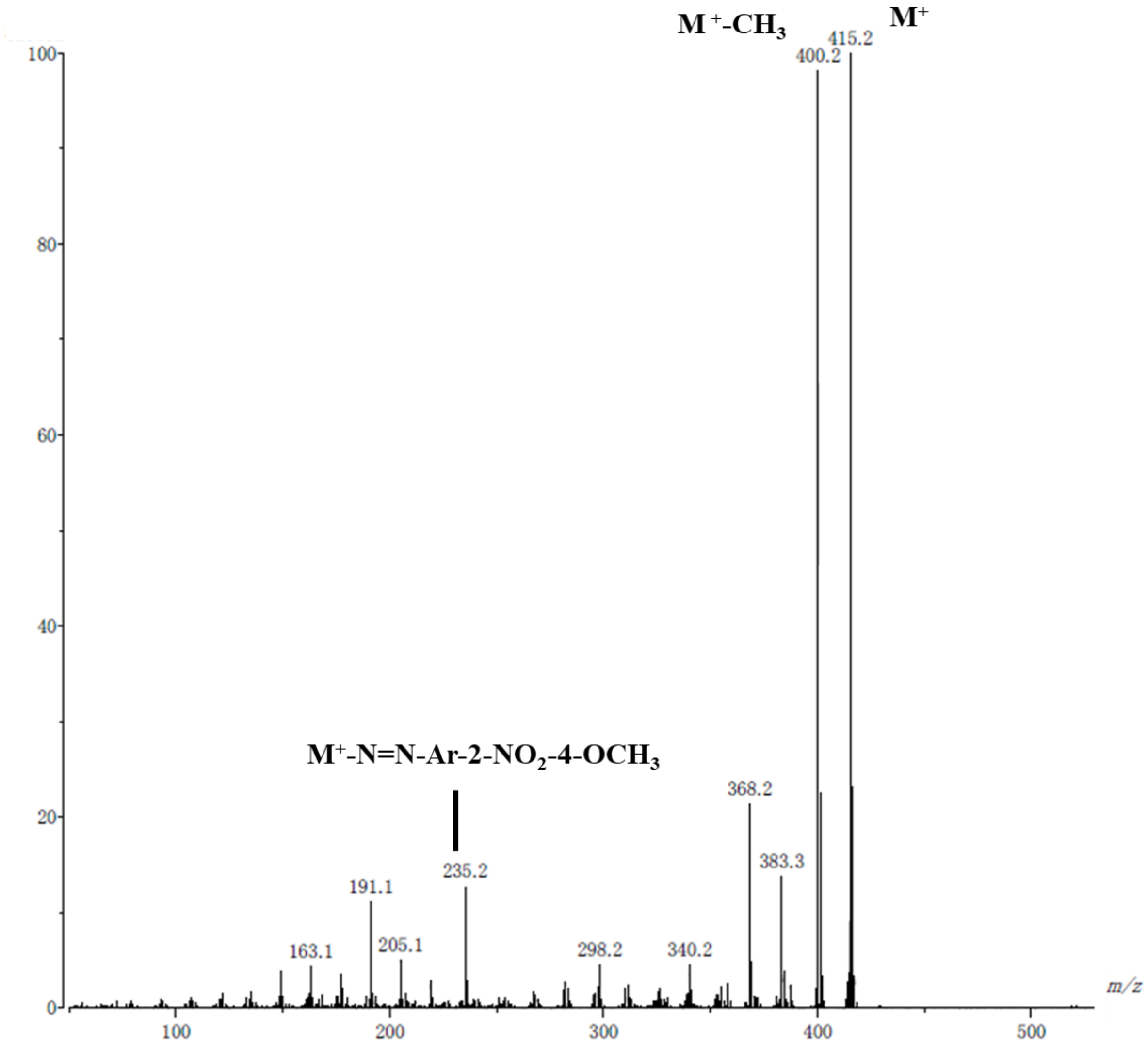

Figure S6. Dye X-377-2-D mass spectroscopy analysis.

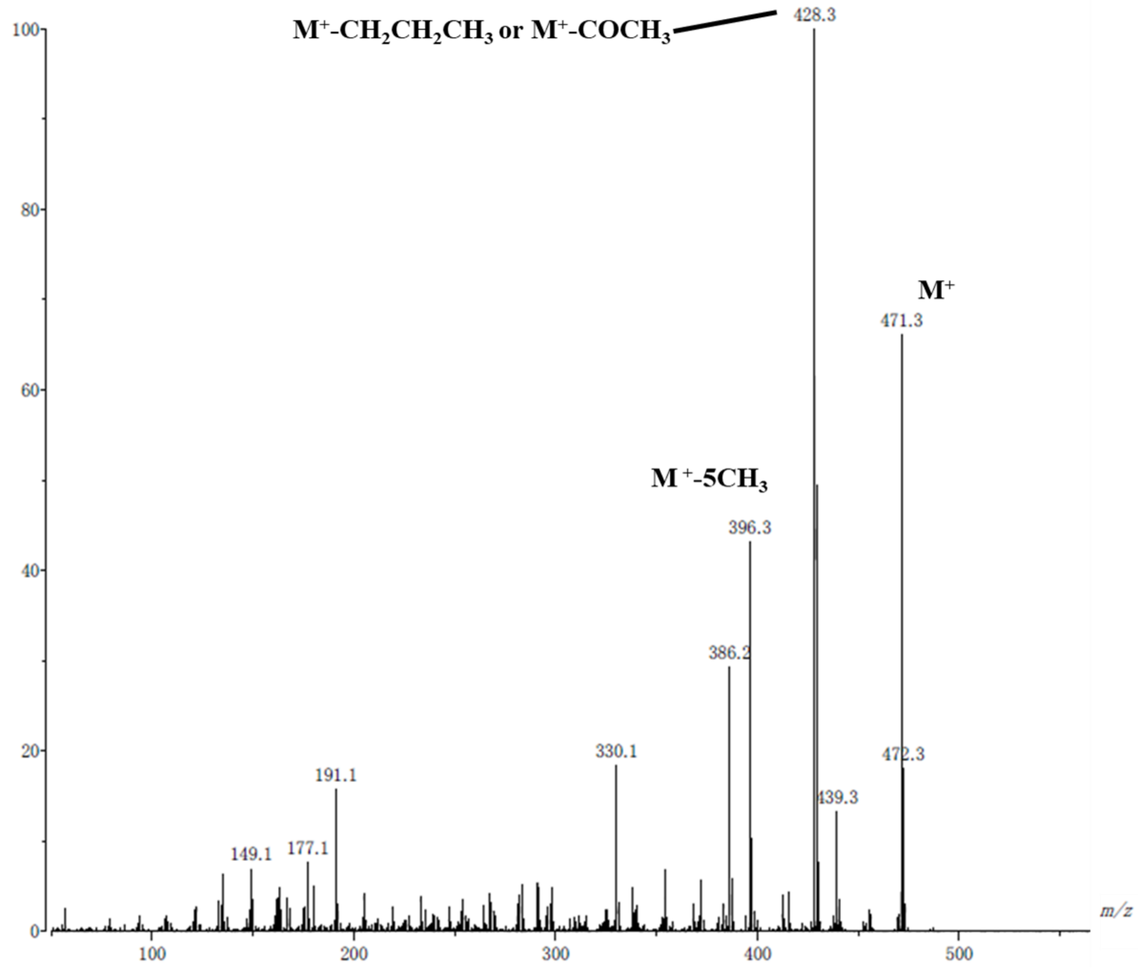

Figure S7. Dye X-377-4-D mass spectroscopy analysis.

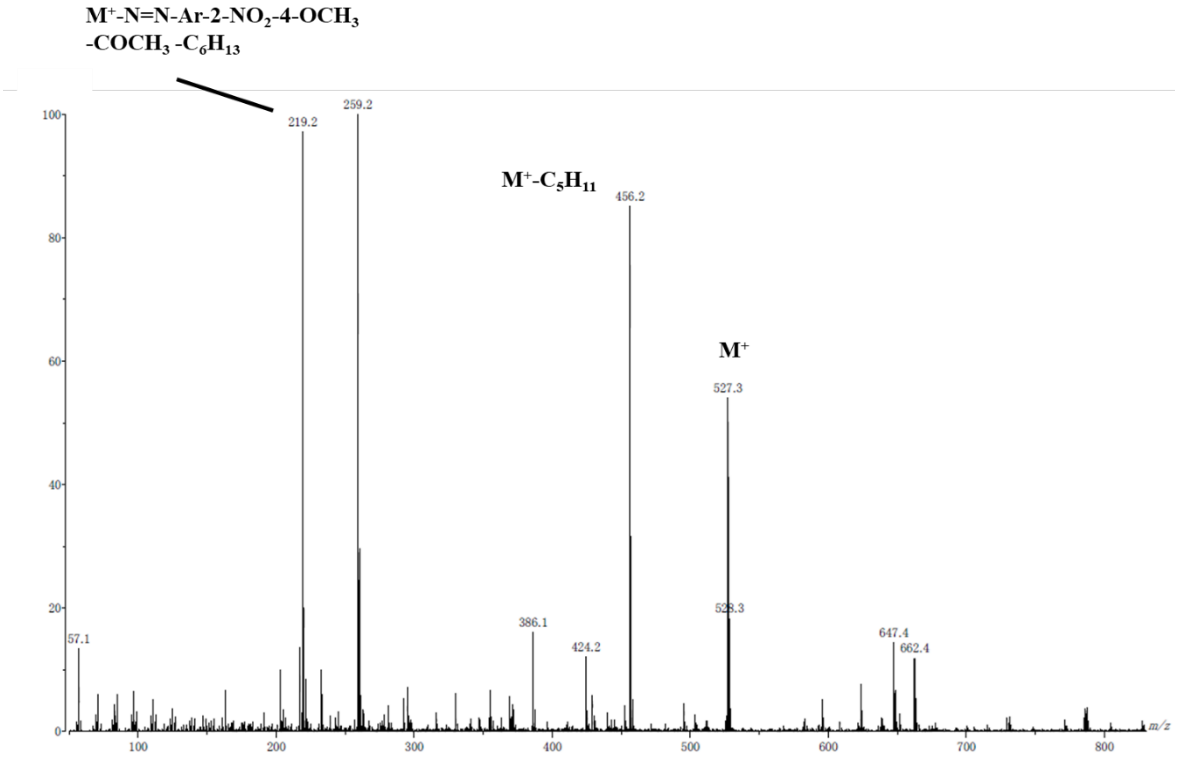

Figure S8. Dye X-377-6-D mass spectroscopy analysis.

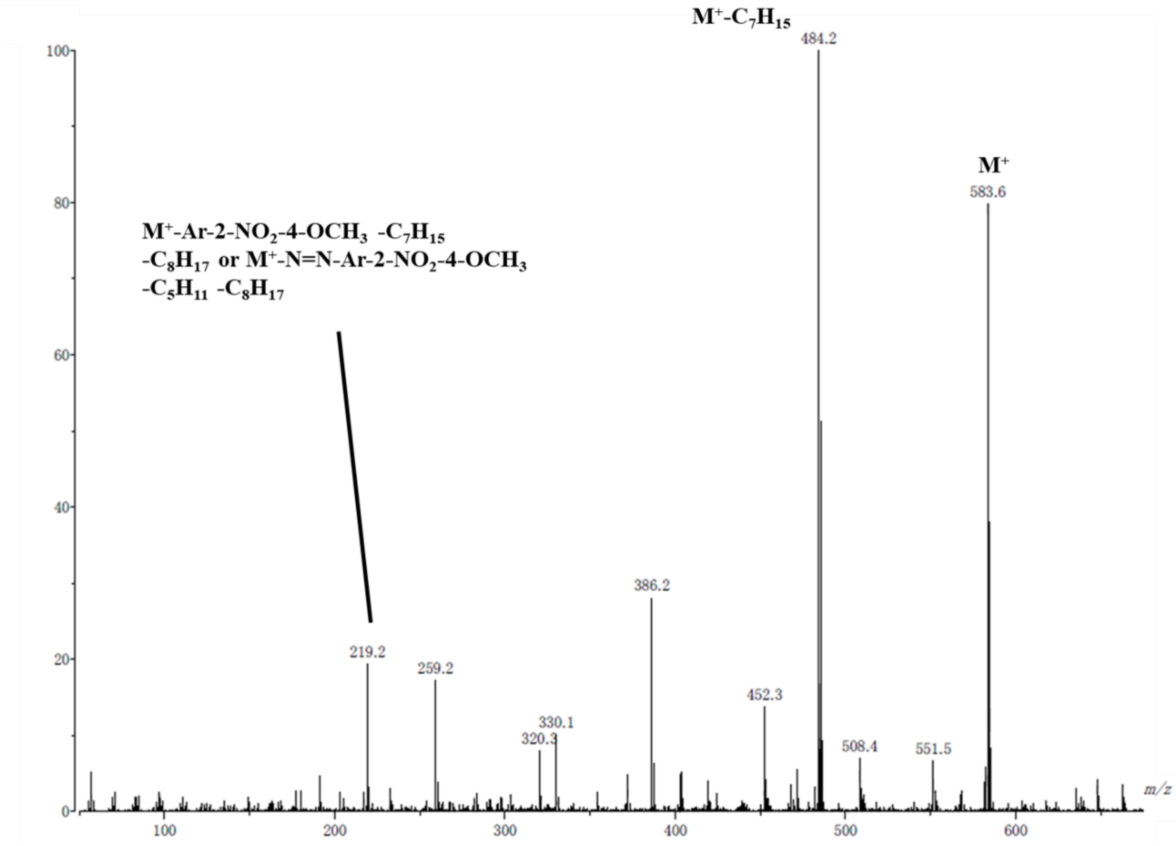

Figure S9. Dye X-377-8-D mass spectroscopy analysis.

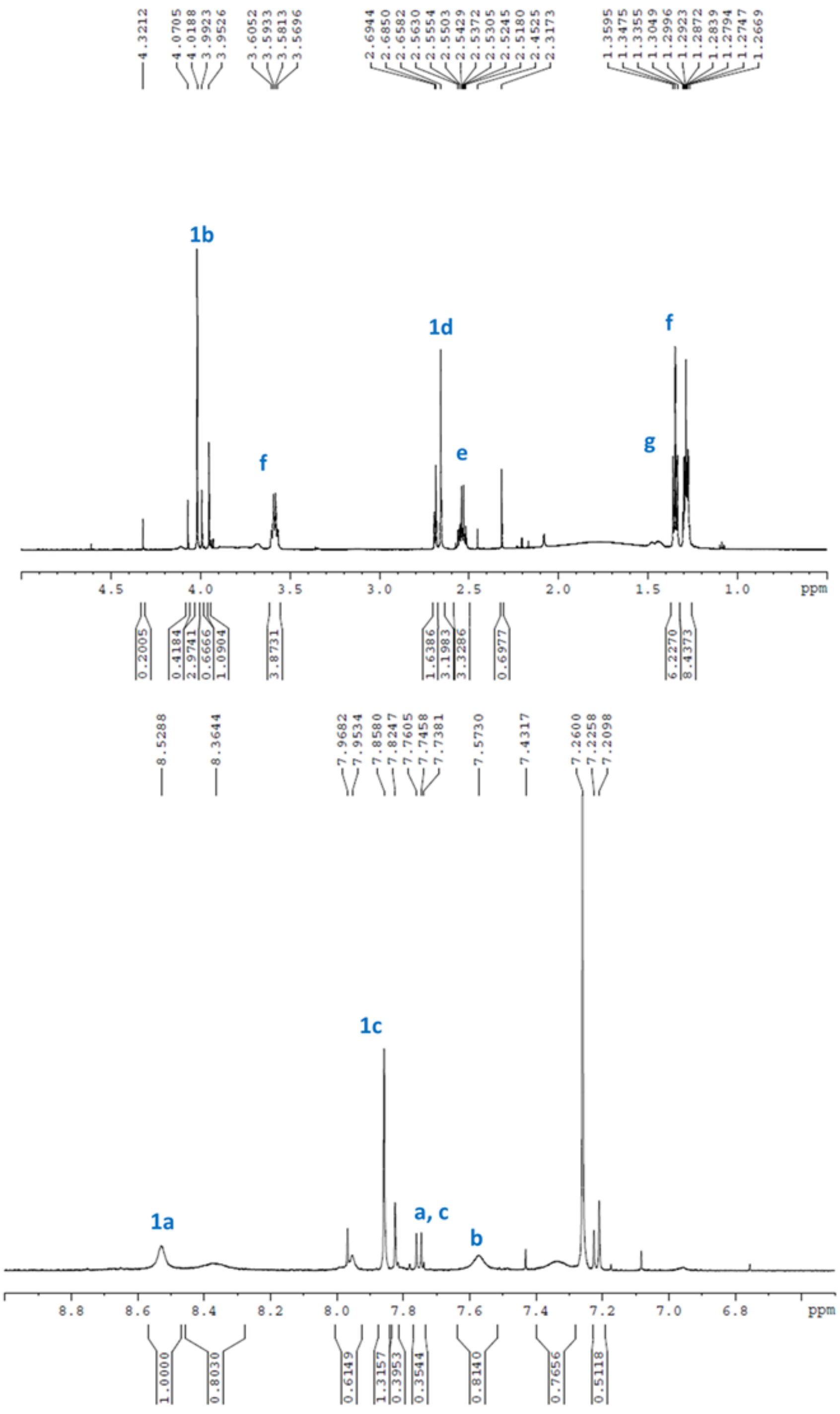

Figure S10. Dye 161-A ^{1}H-NMR analysis in $CDCl_3$.

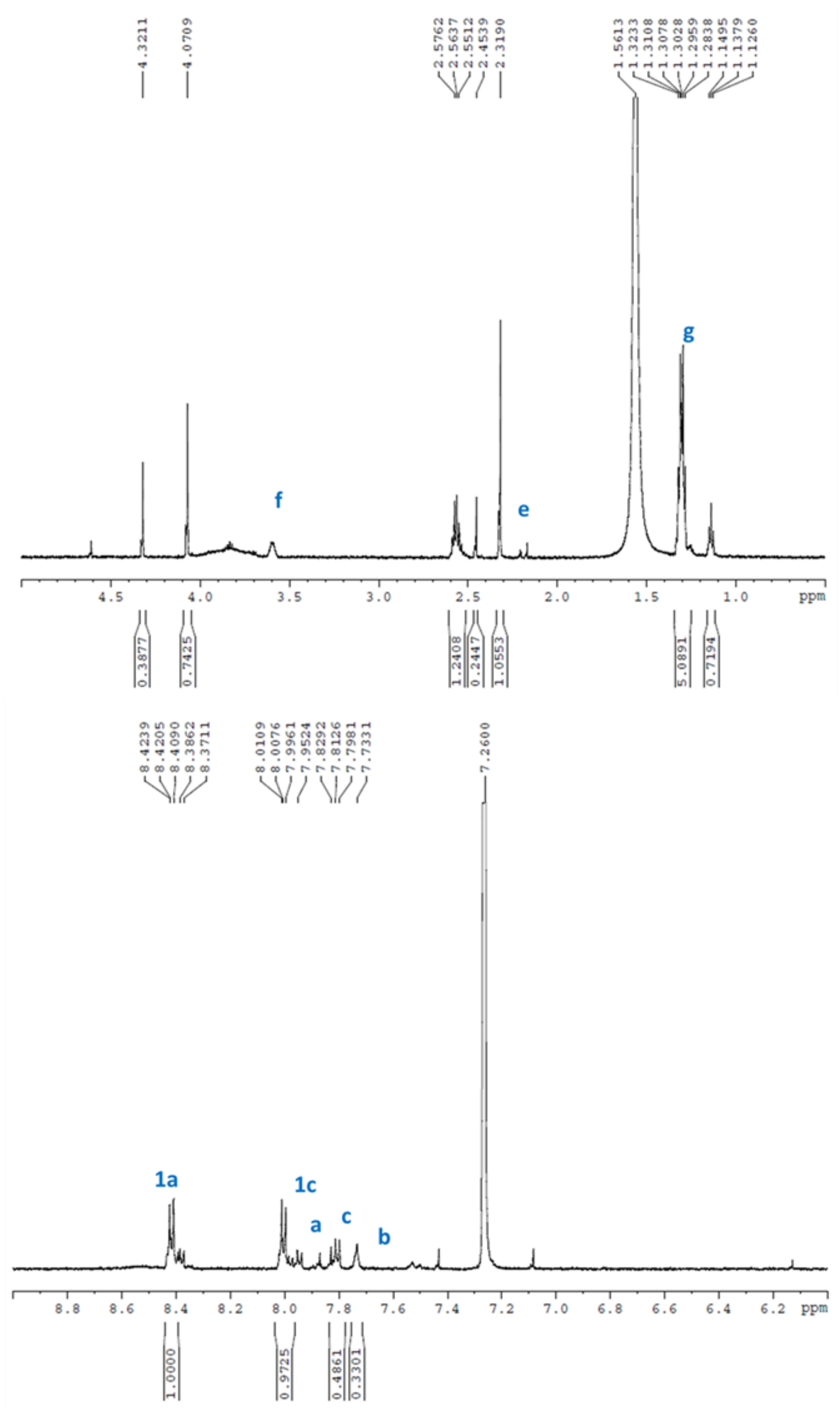

Figure S11. Dye 161-B ^{1}H-NMR analysis in $CDCl_3$.

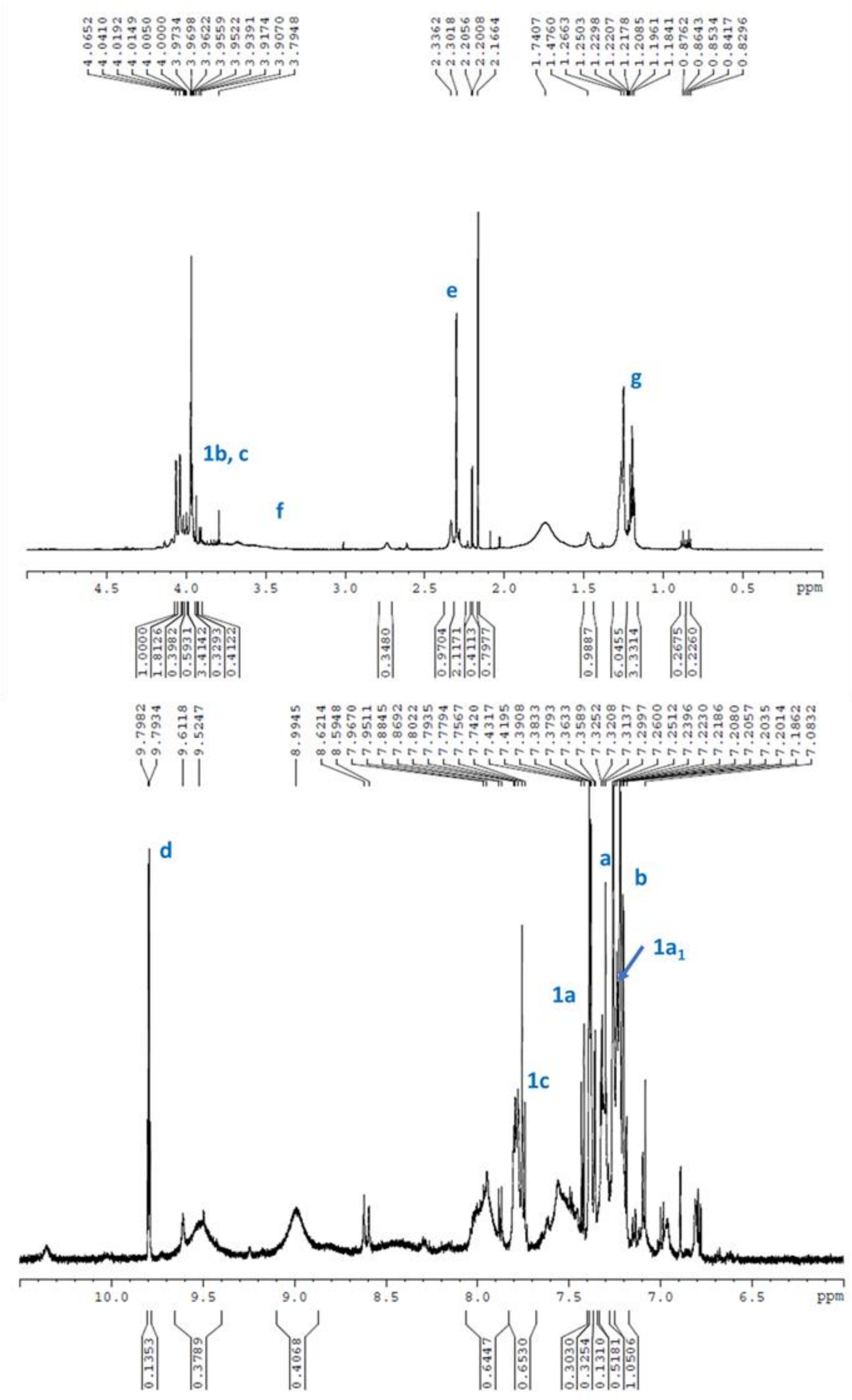

Figure S12. Dye X-377-2-D ^{1}H-NMR analysis in $CDCl_3$.

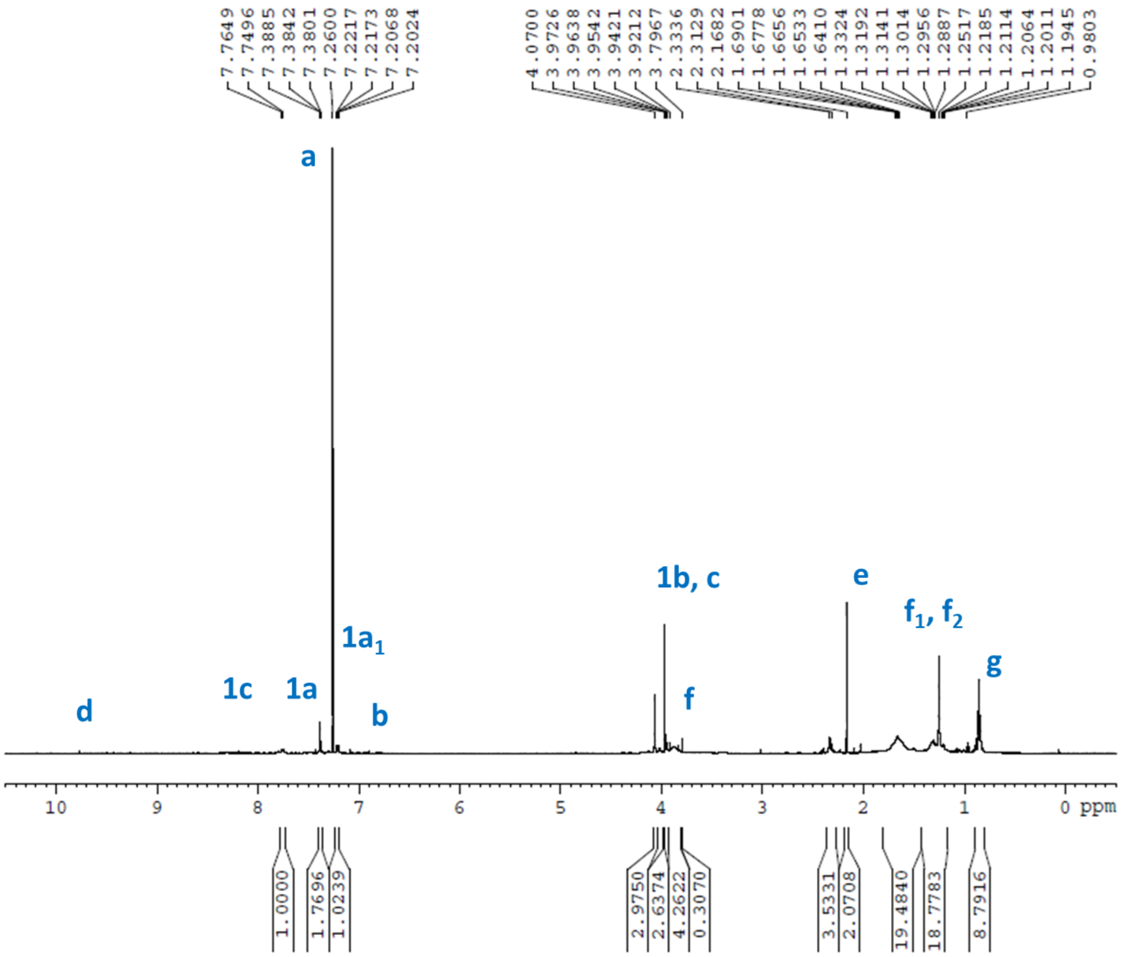

Figure S13. Dye X-377-4-D ^{1}H-NMR analysis in $CDCl_3$.

.

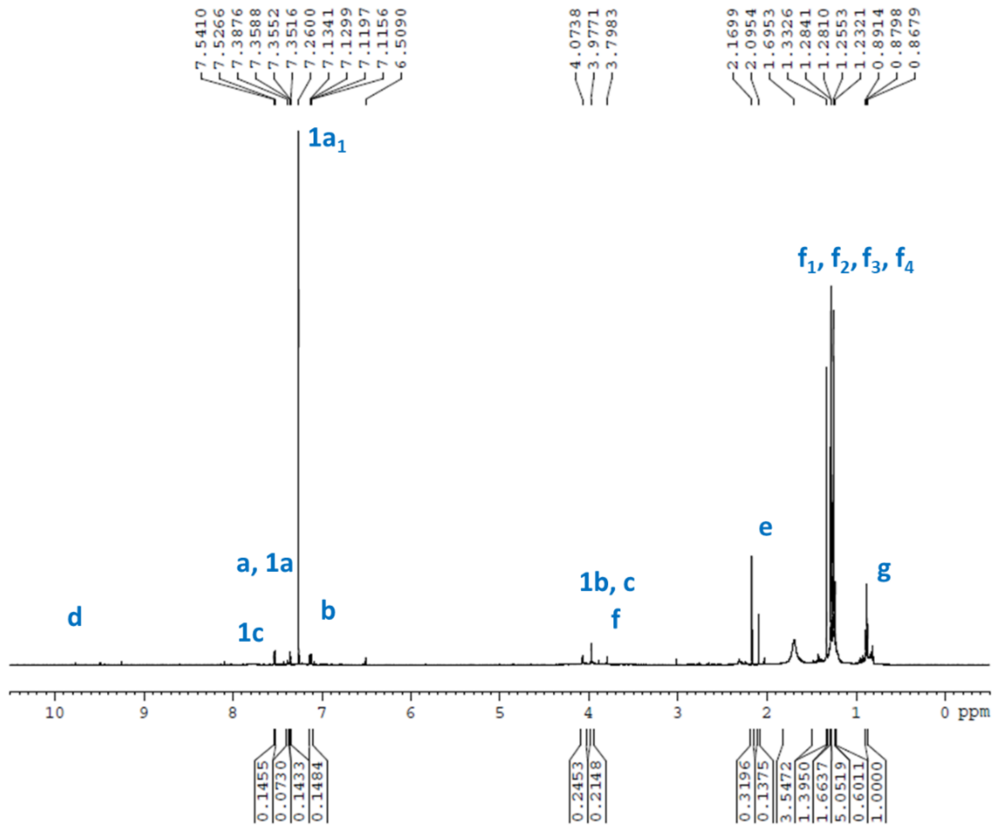

Figure S14. Dye X-377-6-D ^{1}H-NMR analysis in $CDCl_3$.

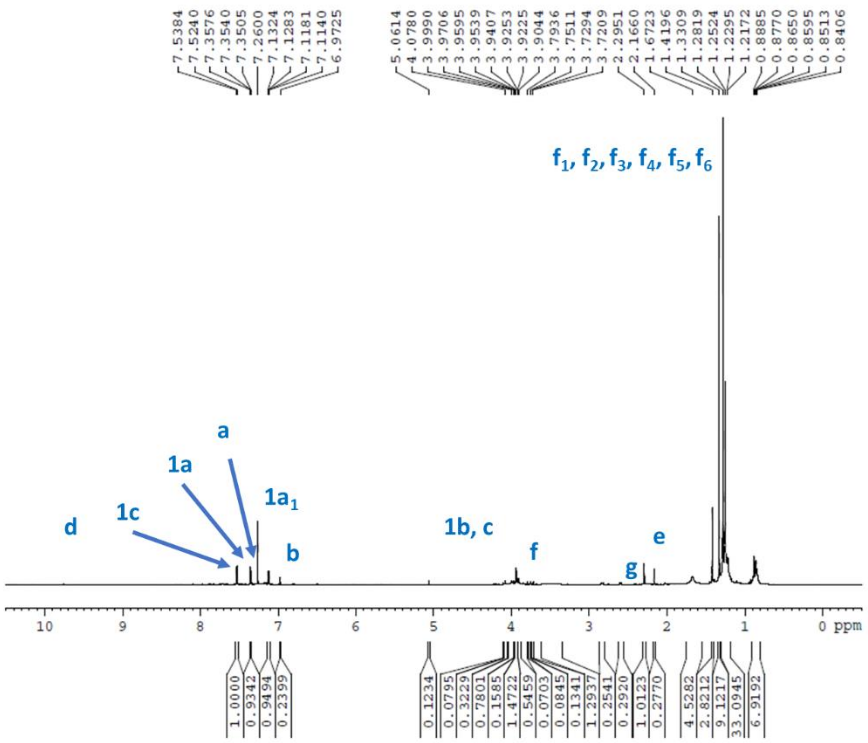

Figure S15. Dye X-377-8-D ^{1}H-NMR analysis in $CDCl_3$.

Table S1. Experimental parameters of disperse dye synthesis.

Dye	Diazo Component (g)	HCl (mL)	H_2O (mL)	Sodium Nitrate (g)	Coupling Component (g)	HAc (mL)	H_2O (mL)
161-A	2.61	6	10	0.73	1.8	7.5	2.5
161-B	0.93	12	20	0.80	2.06	7.5	2.5
X-377-2-D	1.07	7	10	0.81	2.06	7.5	2.5
X-377-4-D	1.21	6	10	0.81	2.06	7.5	2.5
X-377-6-D	1.49	6	10	0.79	2.06	7.5	2.5
X-377-8-D	1.77	6	10	0.81	2.06	7.5	2.5

Table S2. Mass spectrum analysis of dye 161 series.

161-A	M^+	M-CH_3	M-$COCH_3$
	399.2 (100 %)	384.2 (55 %)	369.3 (40 %)
161-B	M^+	M-2CH_3	M-$COCH_3$
	355.2 (100 %)	340.1 (50 %)	325.2 (37 %)

Table S3. Mass spectrum analysis of dye X-377-X-D series.

377-2-D	M^+	M^+-CH_3	M^+-N=N-Ar-2-NO_2-4-OCH_3
	415.2 (100 %)	400.2 (98 %)	235.2 (15 %)
377-4-D	M^+	M^+-$CH_2CH_2CH_3$ or M^+-$COCH_3$	M^+-5(CH_3)
	471.3 (67 %)	428.3 (100 %)	396.3 (43 %)
377-6-D	M^+	M^+-C_5H_{11}	M^+-N=N-Ar-2-NO_2-4-OCH_3-$COCH_3$ -C_6H_{13}
	527.3 (57 %)	456.2 (85 %)	219.2 (97 %)
377-8-D	M^+	M^+-C_7H_{15}	M^+-Ar-2-NO_2-4-OCH_3-$C_7H_{15}C_8H_{17}$ or<br>M^+-N=N-Ar-2-NO_2-4-OCH_3-C_5H_{11} -C_8H_{17}
	583.6 (80 %)	484.2 (100 %)	219.2 (20 %)

Table S4. Disperse dye series NMR analysis.

Dye	δ (ppm)
161-A	δ1.335-1.359(6H,t,H-f,H-g); δ2.5372(3H,s,H-e); δ2.6582(3H,s,H-1d); δ3.569-3.605(4H,q,H-f); δ4.018(3H, s, H-1b); δ7.573(1H, s, H-b); δ7.738-7.745 (2H,d, H-a, H-c); δ7.858(1H, s, H-1c); δ8.528 (1H, s, H-1a)
161-B	δ1.1.302-1.310(6H,t,H-g); δ2.3190(3H,s,H-e); δ3.569-3.605(4H,q,H-f); δ7.733(1H, s,H-b); δ7.798-7.829(2H,d,H-a,H-c); δ8.007-8.010(2H,d,H-1c); δ8.420-8.423(2H,d,H-1a)
X-377-2-D	δ = 1.184-1.196 (t, 6H, N-CH_3, g); δ = 2.301 (s, 3H, $COCH_3$, e); δ = 3.400-3.480(q, 4H, N-CH_2, f); δ = 3.962 (s, 6H, OCH_3, 1b, c); δ = 7.201 (s, 1H, ArH, b); δ = 7.218-7.223 (d-d, 1H, ArH, $1a_1$); δ = 7.320 (s, 1H, ArH, a); δ = 7.358-7.363 (s-d, 1H, ArH, 1a); δ = 7.742-7.756 (d, 1H, ArH, 1c); δ = 9.793 (s, 1H, NH, d)
X-377-4-D	δ = 0.841-0.865 (t, 6H, N-CH_3, g); δ = 1.288-1.314 (m, 8H, N-CH_2, f1, f2); δ = 2.168 (s, 3H, $COCH_3$, e); δ = 3.750-3.781 (t, 4H, N-CH_2, f); δ = 3.955 (s, 6H, OCH_3, 1b, c); δ = 7.102 (s, 1H, ArH, b); δ = 7.202-7.221 (d-d, 1H, ArH, $1a_1$); δ = 7.341 (s, 1H, ArH, a); δ = 7.380-7.384(s-d, 1H, ArH, 1a); δ = 7.749-7.764 (d, 1H, ArH, 1c); δ = 9.782 (s, 1H, NH, d)
X-377-6-D	δ = 0.867-0.891 (t, 6H, N-CH_3, g); δ = 1.232-1.281 (m, 16H, N-CH_2, f1, f2, f3, f4); δ = 2.169 (s, 3H, $COCH_3$, e); δ = 3.750-3.781 (t, 4H, N-CH_2, f); δ = 3.977 (s, 2H, OCH_3, 1b, c); δ = 7.081 (s, 1H, ArH, b); δ = 7.115-7.134 (d-d, 1H, ArH, $1a_1$); δ = 7.351-7.358 (s-d, 2H, ArH, a, 1a); δ = 7.526-7.541(d, 1H, ArH, 1c); δ = 9.792 (s, 1H, NH, d)
X-377-8-D	δ = 0.859-0.877 (t, 6H, N-CH_3, g); δ = 1.217-1.419 (m, 24H, N-CH_2, f1, f2, f3, f4, f5, f6); δ = 2.166 (s, 1H, $COCH_3$, e); δ = 3.753-3.724 (t, 4H, N-CH_2, f); δ = 3.940 (s, 6H, OCH_3, 1b, c); δ = 6.972 (s, 1H, ArH, b); δ = 7.114-7.132 (d-d, 1H, ArH, $1a_1$); δ = 7.354 (s, 1H, ArH, a); δ = 7.350-7.357 (s-d, 1H, ArH, 1a); δ = 7.524-7.538 (d, 1H, ArH, 1c); δ = 9.787 (s, 1H, NH, d)

Table S5. Washing fastness to detergent after dyeing according to the ISO 105 C06:2010 protocol.

Dye	Cellulose Acetate	Cotton	Nylon	Polyester	Acrylic	Wool
161-A	2–3	4	2	3	4	4
161-B	3	4–5	2–3	4	4–5	4–5
X377-2-D	2–3	4–5	2	4	4–5	4–5
X377-4-D	4	4–5	2	4	4–5	4
X377-6-D	4–5	4–5	2–3	4–5	4–5	4–5
X377-8-D	4–5	4–5	4	4–5	4–5	4–5

Table S6. Color fastness in alkali media after dyeing according to the ISO 105 E04:2008 protocol.

Dye	Cellulose acetate	Cotton	Nylon	Polyester	Acrylic	Wool
161-A	4–5	4–5	4	4–5	5	5
161-B	4–5	4–5	4–5	5	5	5
X377-2-D	4–5	4–5	4	4–5	5	5
X377-4-D	4–5	4–5	4	4–5	5	4–5
X377-6-D	4–5	4–5	4–5	5	5	5
X377-8-D	5	5	5	5	5	5
